# Blastocyst quality and perinatal outcomes of frozen-thawed single blastocyst transfer cycles

**DOI:** 10.3389/fendo.2022.1010453

**Published:** 2022-10-12

**Authors:** Nan Jia, Haoying Hao, Cuilian Zhang, Juanke Xie, Shaodi Zhang

**Affiliations:** ^1^Reproductive Medicine Center, Henan Provincial People’s Hospital, ZhengZhou, China; ^2^People’s Hospital of ZhengZhou University, People’s Hospital of Henan University, ZhengZhou, China

**Keywords:** blastocyst quality, perinatal outcomes, frozen-thawed cycle, single blastocyst transfer, singleton live birth

## Abstract

**Objective:**

To investigate the effects of blastocyst quality and morphological grade on the perinatal outcomes in patients undergoing frozen-thawed single blastocyst transfer cycles.

**Methods:**

This single-center retrospective cohort study included 2648 singleton neonates resulting from frozen-thawed single blastocyst transfers performed between January 2017 and September 2021. Multivariate logistic regression was performed to evaluate perinatal outcomes for their association with blastocyst quality and morphological parameters.

**Result:**

Transfer of a good-quality blastocyst in a frozen-thawed cycle was associated with a lower rate of preterm delivery (PTD, adjusted OR =0.7, 95% CI 0.5-0.9; P=0.020) and a higher likelihood of a male neonate (adjusted OR =1.2, 95%CI 1.0-1.5; P=0.048). Compared with grade C inner cell mass (ICM) blastocyst transfer, grade B ICM (adjusted OR =0.5, 95%CI 0.2-0.9; P=0.027) and grade A ICM (adjusted OR =0.6, 95%CI 0.3-1.5; P=0.290) blastocyst transfers were associated with a lower rate of PTD, which was more evident for grade B ICM. After adjusting for confounders, the likelihood of a male neonate (grade B TE, OR =1.2, 95%CI 1.0-1.5, P=0.037; grade A TE OR =1.9, 95%CI 1.3-28, P=0.002) increased with increasing trophectoderm (TE) quality. Compared with expansion stage 4, the likelihood of a male neonate was 1.5 times greater with transfer of a stage 6 blastocyst (OR =1.5, 95%CI 1.0-2.3; P=0.06), and the risk of small for gestational age (SGA) was greater with transfer of a stage 5 blastocyst (adjusted OR =3.5, 95%CI 1.5-8.0; P=0.004). The overall grading of the blastocyst, expansion stage, ICM grade, and TE grade were not associated with length at birth, birthweight, large for gestational age (LGA), or birth defects (all P>0.05).

**Conclusions:**

In frozen-thawed single blastocyst transfer cycles, transfer of a good-quality blastocyst was associated with a lower rate of PTD and a greater likelihood of a male neonate. Transfer of grade B ICM blastocysts decreased the rate of PTD, and TE quality was positively correlated with the likelihood of a male neonate.

## Introduction

With the development of various technologies for *in vitro* fertilization and embryo transfer (IVF-ET), including cryopreservation techniques, and embryo culture systems (optimized culture media and culture conditions), use of selective single blastocyst transfer (SBT) has become more widespread in the field of assisted reproductive technology (ART) (1). Extending the duration of embryo culture to the blastocyst stage offers the opportunity to select the most viable embryos and improves synchronization with the endometrium (2). Compared with cleavage-stage embryo transfer, SBT has resulted in higher clinical pregnancy rate, higher live birth rates, and a lower risk of ectopic pregnancy; it has also prevented multiple pregnancies and decreased the risk of maternal and fetal complications (3, 4). Therefore, selection of good-quality blastocysts for transfer is critical for the better perinatal outcomes.

During recent decades, evidence has emerged demonstrating that the morphological grade of embryos, including blastocysts, correlates with clinical outcomes (5, 6). However, few studies have focused on the association of overall grading and morphological parameters of blastocysts with perinatal outcomes, which remains controversial. Some of the studies have compared fresh cycles with freeze-thawed cycles (3, 7), cleavage-stage embryo transfers with blastocyst transfers (8), and cleavage-stage embryo transfers of good quality with those of poor quality (9, 10). A systematic review showed the increased maternal and fetal health risks were associated with ART treatments (11). Furthermore, researchers have reported that poor-quality blastocyst transfers may result in lower mean birthweight (12). Controversially, several other studies found no association between the quality of the blastocyst and perinatal outcome (13, 14). Therefore, further studies are necessary to confirm the safety of SBT for both mothers and their babies according to blastocyst quality.

In addition, hormonal stimulation of the controlled ovarian hyperstimulation in fresh cycles causes a state of hypoestrogenism, which may result in abnormal angiogenesis in the endometrium, leading to failure of implantation and abnormal placentation (15). Compared with fresh cycles, frozen SBT has resulted in a higher singleton live birth rate, but may also increase the risk of pre-eclampsia (16, 17). Moreover, maternal body mass index (BMI) is related to neonatal birthweight (18) and the incidence of LGA (19).

Therefore, we performed this retrospective cohort study that restricted maternal age and BMI to specific ranges (12, 20) in a single tertiary academic medical center to investigate further the correlation between blastocyst quality, each morphologic characteristic, and a range of perinatal outcomes, including the incidence of PTD, the likelihood of a male neonate, birthweight and birth defects.

## Materials and methods

### Study design and patients

This retrospective cohort study was conducted at the Reproductive Medicine Center of Henan Provincial People’s Hospital affiliated with Zhengzhou University and included patients with a singleton live birth after frozen-thawed SBT between January 2017 and September 2021.

The following inclusion criteria were applied (1): a frozen-thawed SBT cycle (2); age 20-40 years (3); maternal BMI 18-30 kg/m^2^; and (4) a singleton live birth.

The exclusion criteria were as follows (1): cycle in which a stage 3 or lower blastocyst was transferred (2); a congenital or acquired uterine anomaly (3); monozygotic twins; and (4) a pregnancy-related disorder (e.g., pregnancy-induced hypertension). Only data for the first neonate born to patients who delivered twice or more during the study period were included. We collected data from our electronic medical record system for all patients who underwent conventional *in vitro* fertilization and embryo transfer without additional intervention.

### Blastocyst cryopreservation and quality assessment

Laser artificial shrinkage was used before freezing, and the protocol for vitrification and warming (Vitrification Kit, Thawing Kit, Kitazato, Tokyo, Japan) was performed according to the manufacturer’s instructions. Briefly, blastocysts were cryopreserved individually using a Cryotop carrier system with cryoprotectants and transferred into a successively descending concentration gradient solution for thawing. The warmed blastocysts were then cultured for 2-4 hours in a desktop incubator (Cook Medical, Bloomington, IN, USA), and the blastocyst with the most favorable survival signs (≥75% of blastomeres showing no signs of damage) (21) was transferred.

According to our procedure, at least two experienced embryologists assessed the blastocysts independently according to the Gardner and Schoolcraft grading system (22). The score depends mainly on three morphological parameters: blastocyst expansion, ICM, and TE. Due to the transfer strategy used at our center, a few cycles of expansion stage 3 blastocyst transfers had a singleton live birth during the study period. Stage 3 transfers were then excluded to reduce sampling bias. The blastocysts were divided into three groups: stage 4 (an expanded blastocyst with a thinning zona pellucida); stage 5 (a hatching blastocyst with TE starting to protrude through the zona pellucida); and stage 6 (the hatched blastocyst completely detached from the zona pellucida). Blastocysts with an expansion stage >3, ICM grade, and TE grade higher than C (≥ 4BB) were considered good quality (23). The remaining blastocysts (with ICM and TE grades that were not graded C simultaneously) were defined as poor-quality blastocysts. The ICM was scored as follows: (A) many tightly packed cells; (B) composed of several cells grouped loosely; or (C) very few loosely arranged cells. TE was graded as follows: (A) many cells that form a cohesive epithelium; (B) few cells that form a loose epithelium; or (C) very few cells that have difficulty forming cohesive epithelia. Our center has obtained ISO9001 certification for quality assessment, and the blastocysts were scored by two of five experienced embryologists. In cases of disagreement, the other three embryologists would provide their opinions.

### Preparation of the endometrium and luteal support

Details of the endometrial preparation and luteal support procedures used at our center have been described elsewhere (24). In brief, two methods were prepared, namely, a natural cycle for patients with regular menstrual cycles and a hormone replacement cycle for patients with irregular menstruation (or a history of thin endometrium). Blastocysts were transferred on day 6 after administration of progesterone, and luteal support was continued until 10 weeks if pregnancy was achieved.

### Outcome definitions and statistical analysis

Perinatal outcomes included PTD (delivery before 37 weeks’ gestation), length at birth, sex of the neonate, absolute birthweight, low birthweight (LBW, birthweight <2500 g at any gestational week), macrosomia (birthweight >4000 g at any gestational week), LGA (birthweight >90th percentile), SGA (birthweight <10th percentile), and birth defects. LGA and SGA were adjusted for gestational age and sex and calculated based on data for Chinese singletons (25).

Normally distributed and skewed continuous variables are expressed as the mean and standard deviation or as the median and interquartile range. Data were compared between groups using the independent-samples *t*-test, Kruskal-Wallis test, chi-square test, or Fisher’s exact test as appropriate. Multivariable logistic regression analysis was performed to estimate the odds ratio (OR) or beta coefficient (β) and 95% confidence interval (CI) for perinatal outcomes according to blastocyst quality and morphologic grade. All statistical analyses were performed using R language-based software (Empower Stats, Greenwood Village, CO, USA). A P-value < 0.05 was considered statistically significant.

## Results

### Baseline characteristics

**Figure 1:**
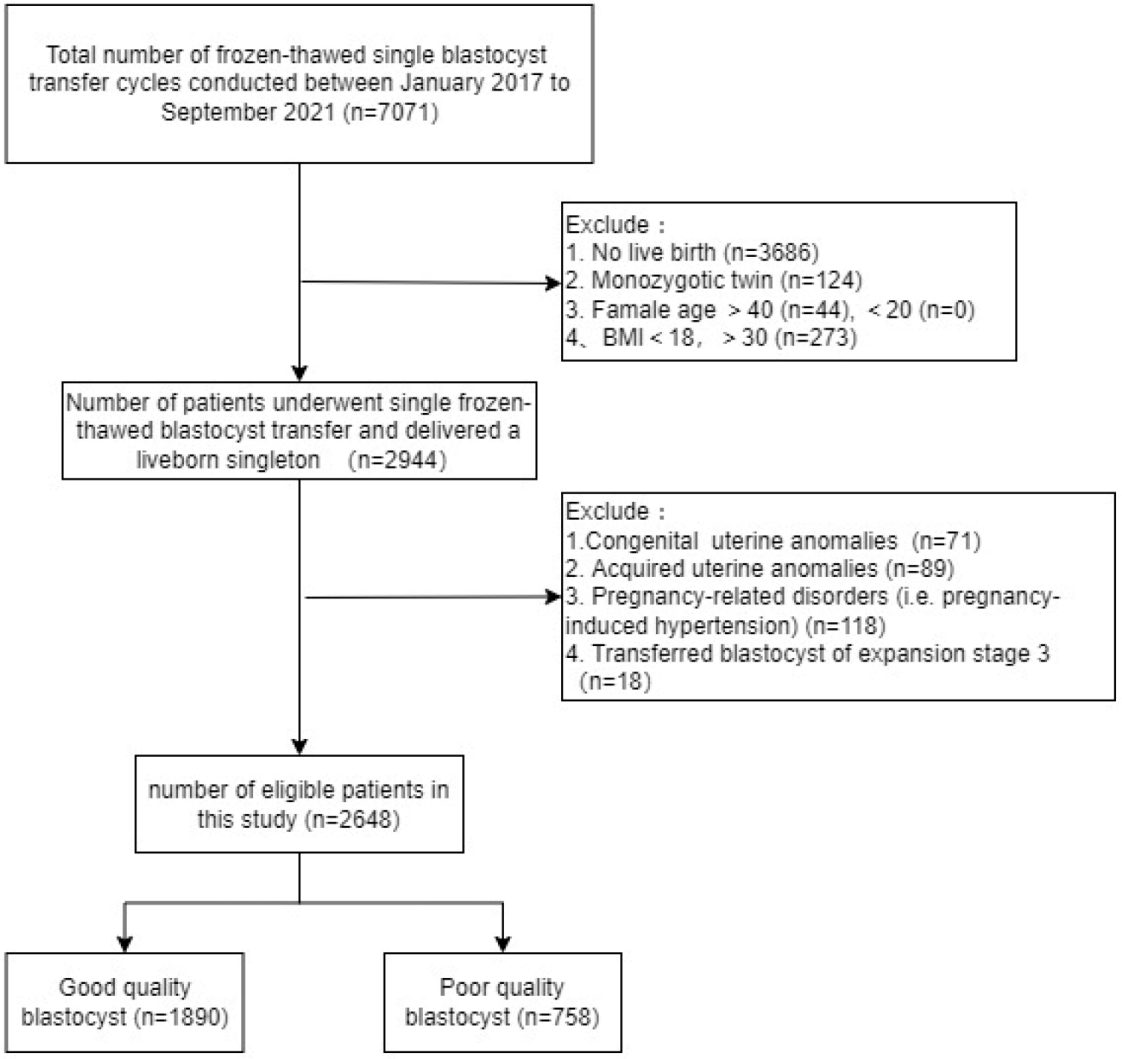
Flow chart of the study.

We identified 7071 patients who underwent frozen-thawed SBT, 2648 of whom met the eligibility criteria and were included in the analysis. According to the definitions of good-quality and poor-quality blastocysts described in the Methods section, 1890 (71.37%) cycles transferred a good-quality blastocyst and 758 (28.63%) transferred a poor-quality blastocyst. The specific flowchart is shown in **Figure 1**.

The distribution of maternal age (P<0.001), basal anti-Müllerian hormone (AMH) level (P=0.004), number of 2PN (P<0.001), fertilization method (P<0.001), transfer cycle rank (P<0.001), parity (P=0.038), and transfer day (P<0.001) differed significantly according to blastocyst quality category. However, there was no statistically significant difference in terms of maternal BMI (P=0.327), downregulation method used (P=0.187), duration of infertility (P=0.514), primary infertility ratio (P=0.211), endometrial thickness (P=0.804), or type of endometrial hormone replacement used (P=0.648). The patient characteristics are shown in **Table 1**.

**Table 1:** Characteristics of patients with good or poor quality blastocyst transfer.

	Poor-quality blastocyst (n = 758)	Good-quality blastocyst (n = 1890)	P value
Female age (years)	30.83 ± 4.08	30.21 ± 3.75	<0.001
maternal BMI (kg/m^2^)	22.85 ± 2.83	22.73 ± 2.81	0.327
Baseline AMH (ng/ml)	4.15 (2.38-6.82)	4.87 (3.01-7.47)	0.004
Number of 2PN	8.00 (6.00-11.00)	10.00 (8.00-14.00)	<0.001
Fertilization method			<0.001
IVF	474 (62.53%)	1393 (73.70%)	
ICSI	255 (33.64%)	442 (23.39%)	
IVF+ICSI	29 (3.83%)	55 (2.91%)	
Downregulation method			0.187
long agonist protocol	601 (81.44%)	1528 (83.50%)	
GnRH-antagnist project	101 (13.69%)	204 (11.15%)	
The other project	36 (4.88%)	98 (5.36%)	
Duration of infertility (years)	3.00 (1.50-4.50)	3.00 (1.50-4.00)	0.514
Primary infertility	357 (47.10%)	941 (49.79%)	0.211
Transfer cycle rank			<0.001
1	368 (48.55%)	1236 (65.40%)	
2	272 (35.88%)	522 (27.62%)	
High order	118 (15.57%)	132 (6.98%)	
Parity>1(%)	176 (23.25%)	371 (19.64%)	0.038
Endometrial preparation of HRT	638 (84.73%)	1606 (85.43%)	0.648
Endometrium thickness (mm)	9.74 ± 1.77	9.76 ± 1.79	0.804
Transfer day			<0.001
D5	421 (55.54%)	1492 (78.94%)	
D6	337 (44.46%)	398 (21.06%)	

BMI, body mass index; AMH, anti-Müllerian hormone; PN, pronucleus; IVF, in vitro fertilization; ICSI, Intracytoplasmic sperm injection; HRT, hormone replaced cycle.

*X^2^* or t-test as appropriate. Data are expressed with mean (SD) or number (percent) as appropriate.

### Perinatal outcomes according to quality of blastocyst transferred

Perinatal outcomes stratified by blastocyst quality are presented in **Table 2**. In total, there were 1086 cycles (57.46%) resulting in male infants undergoing a good-quality SBT, and the difference was statistically significant compared with poor-quality SBT (P=0.009). Multiple regression analysis adjusted for multiple variables showed that good-quality blastocyst transfer was associated with a lower rate of PTD (adjusted OR =0.7, 95%CI 0.5-0.9) and an increased rate of male neonates (adjusted OR =1.2, 95%CI 1.0-1.5). There was no statistically significant between-group difference in length at birth (P=0.915), frequency of birth defects (P=0.290), birthweight (P=0.145), or related classifications (LBW, P=0.423; macrosomia, P=0.785; LGA, P=0.958; and SGA, P=0.479).

**Table 2:** Perinatal outcomes of live-born singletons according to quality of blastocyst transferred.

	Poor-quality blastocyst (n = 758)	Good-quality blastocyst (n = 1890)	P value^a^	Good VS Poor Non-adjusted^b^		Good VS Poor Adjusted^b^	
				OR (95%CI)	P value	OR (95%CI)	P value
PTD	67 (8.84%)	126 (6.67%)	0.052	0.7 (0.5, 1.0)	0.053	0.7 (0.5, 0.9)	0.020
length at birth(cm)	50.26 ± 1.68	50.25 ± 1.60	0.938	–0.0 (-0.1, 0.1)	0.938	–0.0 (-0.2, 0.1)	0.915
Male neonate	393 (51.85%)	1086 (57.46%)	0.009	1.3 (1.1, 1.5)	0.009	1.2 (1.0, 1.5)	0.048
Birth defects	4 (0.53%)	12 (0.63%)	0.748	1.2 (0.4, 3.7)	0.748	2.2 (0.5, 9.8)	0.290
Birthweight (g)	3413.39 ± 489.45	3448.79 ± 483.54	0.090	35.4 (-5.5, 76.3)	0.090	33.9 (-11.7, 79.5)	0.145
LBW	21 (2.77%)	41 (2.17%)	0.355	0.8 (0.5, 1.3)	0.356	0.8 (0.4, 1.4)	0.423
Macrosomia	68 (8.97%)	184 (9.74%)	0.545	1.1 (0.8, 1.5)	0.545	1.0 (0.7, 1.5)	0.785
LGA	195 (25.76%)	484 (25.65%)	0.953	1.0 (0.8, 1.2)	0.953	1.0 (0.8, 1.3)	0.958
SGA	21 (2.77%)	60 (3.18%)	0.584	1.2 (0.7, 1.9)	0.585	1.2 (0.7, 2.1)	0.479

OR, odds ratio; PTD, preterm delivery; LBW, low birthweight; LGA, large for gestational age; SGA, small for gestational age

a *X^2^* or t-test as appropriate. Data are expressed with mean ± SD or number (percent) as appropriate.

b Adjusted with female age, maternal body mass index, baseline AMH, number of 2PN, primary infertility, duration of infertility, fertilization method, transfer cycle rank, parity, FET endometrial preparation, endometrium thickness, transfer day.

### Morphological grading and perinatal outcomes

Compared with grade C ICM blastocyst transfer, there was a trend toward a decreased rate of PTD in grade B and grade A ICM blastocyst transfer, and the decline in grade B ICM was more prominent (adjusted OR 0.5, 95% CI 0.2–0.9 for grade B ICM; adjusted OR 0.6, 95% CI 0.3–1.5 for grade A ICM). The higher the TE grade, the higher the male birth rate (adjusted OR 1.2, 95% CI 1.0–1.5 for grade B TE; adjusted OR 1.9, 95% CI 1.3–2.8 for grade A TE), as shown in **Table 3**. The likelihood of a male neonate was higher for expansion stage 6 blastocyst transfer (adjusted OR 1.5, 95% CI 1.0–2.3) and lower for stage 5 blastocyst transfer (adjusted OR 0.8, 95% CI 0.5–1.2) than for stage 4. A singleton neonate resulting from an expansion stage 5 blastocyst was at higher risk of being SGA (adjusted OR 3.5, 95% CI 1.5–8.0) than one resulting from a stage 4 blastocyst transfer. The associations with other perinatal outcomes were not statistically different, and the specific effect sizes are shown in **Tables 3**, **4**.

**Table 3:** Perinatal outcomes of live-born singletons according to blastocyst growth parameters.

	PTD			Length at birth			Male neonate			Birth defects		
	N (%)	Non-adjusted	Adjusted	Mean ± SD	Non-adjusted	Adjusted	N (%)	Non-adjusted	Adjusted	N (%)	Non-adjusted	Adjusted
		OR (95%CI)	OR (95%CI)		β(95%CI)	β(95%CI)		OR (95%CI)	OR (95%CI)		OR (95%CI)	OR (95%CI)
Expansion stage												
4	172 (7.09%)	reference	reference	50.24 ± 1.63	reference	reference	1354 (55.79%)	reference	reference	12 (0.49%)	reference	reference
5	10 (9.90%)	1.4 (0.7, 2.8)	1.6 (0.8, 3.3)	50.26 ± 1.59	0.0 (-0.3, 0.3)	– 0.0 (-0.4, 0.3)	51 (50.50%)	0.8 (0.5, 1.2)	0.8 (0.5, 1.2)	2 (1.98%)	4.1 (0.9, 18.4)	2.7 (0.3, 28.5)
6	11 (9.17%)	1.3 (0.7, 2.5)	1.2 (0.6, 2.5)	50.43 ± 1.57	0.2 (-0.1, 0.5)	0.1 (-0.2, 0.5)	74 (61.67%)	1.3 (0.9, 1.9)	1.5 (1.0, 2.3)	2 (1.67%)	3.4 (0.8, 15.4)	5.0 (0.7, 36.6)
ICM stage												
C	13 (14.94%)	reference	reference	50.05 ± 1.12	reference	reference	52 (59.77%)	reference	reference	1 (1.15%)	reference	reference
B	164 (6.81%)	0.4 (0.2, 0.8)	0.5 (0.2, 0.9)	50.27 ± 1.63	0.2 (-0.1, 0.6)	0.2 (-0.1, 0.6)	1338 (55.54%)	0.8 (0.5, 1.3)	0.8 (0.5, 1.3)	15 (0.62%)	0.5 (0.1, 4.1)	0.5 (0.0, 4.5)
A	16 (10.53%)	0.7 (0.3, 1.5)	0.6 (0.3, 1.5)	50.07 ± 1.74	0.0 (-0.4, 0.5)	0.1 (-0.3, 0.6)	89 (58.55%)	1.0 (0.6, 1.6)	0.9 (0.5, 1.6)	0 (0.00%)	0.0 (0.0, Inf)	0.0 (0.0, Inf)
TE stage												
C	54 (8.05%)	reference	reference	50.28 ± 1.74	reference	reference	341 (50.82%)	reference	reference	3 (0.45%)	reference	reference
B	120 (6.59%)	0.8 (0.6, 1.1)	0.7 (0.5, 1.1)	50.23 ± 1.57	– 0.1 (-0.2, 0.1)	– 0.1 (-0.2, 0.1)	1038 (57.03%)	1.3 (1.1, 1.5)	1.2 (1.0, 1.5)	13 (0.71%)	1.6 (0.5, 5.6)	3.6 (0.6, 20.0)
A	19 (12.10%)	1.6 (0.9, 2.7)	1.3 (0.7, 2.3)	50.38 ± 1.70	0.1 (-0.2, 0.4)	0.2 (-0.2, 0.5)	100 (63.69%)	1.7 (1.2, 2.4)	1.9 (1.3, 2.8)	0 (0.00%)	0.0 (0.0, Inf)	0.0 (0.0, Inf)

OR, odds ratio; PTD, preterm delivery.

Data are expressed with mean ± SD or number (percent) as appropriate.

Adjusted with female age, maternal body mass index, baseline AMH, number of 2PN, primary infertility, duration of infertility, fertilization method, transfer cycle rank, parity, FET endometrial preparation, endometrium thickness, transfer day.

**Table 4:** Association between birthweight of live-born singletons and blastocyst growth parameters.

	Birthweight			LGA			SGA		
	Mean ± SD	Non-adjusted	Adjusted	N (%)	Non-adjusted	Adjusted	N (%)	Non-adjusted	Adjusted
		β(95%CI)	β(95%CI)		OR (95%CI)	OR (95%CI)		OR (95%CI)	OR (95%CI)
Expansion stage									
4	3440.58 ± 486.22	reference	reference	632 (26.08%)	reference	reference	71 (2.93%)	reference	reference
5	3349.31 ± 490.94	– 91.3 (-187.8, 5.3)	– 73.7 (-177.5, 30.1)	18 (17.82%)	0.6 (0.4, 1.0)	0.6 (0.4, 1.1)	8 (7.92%)	2.8 (1.3, 6.1)	3.5 (1.5, 8.0)
6	3475.00 ± 459.25	34.4 (-54.5, 123.4)	50.6 (-47.5, 148.7)	29 (24.17%)	0.9 (0.6, 1.4)	0.9 (0.6, 1.5)	2 (1.67%)	0.6 (0.1, 2.3)	0.3 (0.0, 2.6)
ICM stage									
C	3392.47 ± 491.47	reference	reference	22 (25.29%)	reference	reference	3 (3.45%)	reference	reference
B	3440.36 ± 482.96	47.9 (-56.0, 151.7)	24.7 (-89.2, 138.7)	618 (25.69%)	1.0 (0.6, 1.7)	1.1 (0.6, 2.0)	76 (3.16%)	0.9 (0.3, 3.0)	1.4 (0.3, 6.0)
A	3438.09 ± 521.45	45.6 (-82.3, 173.6)	8.3 (-131.3, 147.9)	39 (25.83%)	1.0 (0.6, 1.9)	1.0 (0.5, 2.1)	2 (1.32%)	0.4 (0.1, 2.3)	0.4 (0.0, 4.2)
TE stage									
C	3416.10 ± 489.49	reference	reference	173 (25.82%)	reference	reference	18 (2.69%)	reference	reference
B	3448.21 ± 480.93	32.1 (-10.9, 75.1)	31.1 (-15.8, 78.0)	465 (25.59%)	1.0 (0.8, 1.2)	1.0 (0.8, 1.2)	58 (3.19%)	1.2 (0.7, 2.0)	1.2 (0.7, 2.1)
A	3424.30 ± 518.26	8.2 (-76.1, 92.5)	33.5 (-57.8, 124.8)	41 (26.11%)	1.0 (0.7, 1.5)	1.1 (0.7, 1.7)	5 (3.18%)	1.2 (0.4, 3.3)	1.2 (0.4, 3.7)

OR, odds ratio; LGA, large for gestational age; SGA, small for gestational age.

Data are expressed with mean ± SD or number (percent) as appropriate.

Adjusted with female age, maternal body mass index, baseline AMH, number of 2PN, primary infertility, duration of infertility, fertilization method, transfer cycle rank, parity, FET endometrial preparation, endometrium thickness, transfer day.

### Birth defects

There were only 16 cycles with birth defects because of the strict criteria used in this study (0.63% for good-quality blastocyst transfer vs 0.53% for poor-quality blastocyst transfer, P=0.748; **Table 2**). The group in which a good-quality blastocyst was transferred had 3 cases of congenital malformation of the eyes/ears/face/neck and 3 cases of congenital malformations of the digestive system. The distribution of birth defects is shown in **Table 5**.

**Table 5:** Distribution of birth defects in live-born singletons according to quality of blastocyst transferred.

	Poor quality blastocyst (n = 758)	Good quality blastocyst (n = 1890)
Any defect	4 (0.53%)	12 (0.63%)
Q00–Q07 Congenital malformations of the nervous system	0	0
Q10–Q18 Congenital malformations of eye, ear, face and neck	1 (0.13%)	3 (0.16%)
Q20-Q28 Congenital malformations of the circulatory system	1 (0.13%)	0
Q30-Q34 Congenital malformations of the respiratory system	0	0
Q35-Q37 Cleft lip and cleft palate	1 (0.13%)	1 (0.05%)
Q38-Q45 Congenital malformations of the digestive system	0	3 (0.16%)
Q50-Q56 Congenital malformations of genital organs	1 (0.13%)	0
Q60-Q64 Congenital malformations of the urinary system	0	2 (0.11%)
Q65-Q79 Congenital malformations of the musculoskeletal system	0	0
Q90-Q99 Chromosomal abnormalities, not elsewhere classified	0	2 (0.11%)
D50-D89 Hematologic abnormalities	0	0
E00-E90 Metabolic abnormalities	0	0
Q80-Q89 Other congenital malformations	0	1 (0.05%)

Data are expressed with number (percent).

## Discussion

This single-center retrospective study aimed to determine whether blastocyst quality and morphologic grade are independently associated with perinatal outcomes after frozen-thawed cycles. We found that transfer of good-quality blastocysts was associated with a lower rate of PTD and a higher likelihood of a male neonate, respectively, particularly in terms of ICM and TE; grade A and grade B ICM blastocyst transfers were associated with a lower rate of PTD, and a higher TE grade was associated with an increased probability of a male neonate. Compared with stage 4, expansion stage 6 was associated with a higher probability of a male neonate, and stage 5 was associated with a higher risk of SGA. However, given the small number of SGA births and the lack of a trend in male neonates in our study, the effect of expansion stage on perinatal outcomes needs confirmation in further studies. There were no statistically significant differences in other perinatal outcome measures, including length at birth, birthweight, LGA, and birth defects, according to the quality of the blastocyst transferred.

As one of the perinatal outcome indicators, PTD also affects neonatal length at birth and birthweight. Our study demonstrated an association between the overall grading of the blastocyst, ICM score, and PTD. To the best of our knowledge, no previous studies have found a decrease in PTD rate of good-quality blastocyst transfers, that was correlated with the ICM score. Huang et al. recently detected a higher PTD rate after transfer of poor-quality cleavage-stage embryos, which was less evident in blastocyst transfer cycles (26). Meanwhile, another study (13) found a positive correlation of ICM grade with PTD; however, the authors could not explain the observation that the better the quality of ICM, the higher the PTD rate. The differences in the findings of these studies may be caused by use of different inclusion and exclusion criteria and use of different methods for grading the morphological parameters of blastocysts.

Furthermore, it has been shown that perinatal outcomes, including length at birth, birthweight, and birth defects, may not be related to blastocyst quality, such as ICM or TE grade (27, 28). These reports are consistent with our present findings. Data from a cohort study (29) that included only natural-cycle frozen euploid blastocyst transfers suggested that blastocyst morphology was not associated with perinatal outcomes such as PTD or LBW. A multicenter study from Denmark (30) showed that expansion stage, TE, and ICM grade were not associated with PTD, birthweight, or length at birth. Therefore, the investigators believed that blastocyst quality should not be used as a predictive marker for fetal growth potential, defined as birthweight and length at birth. Zhang et al. (12) showed that birthweight was associated with blastocyst quality when grouped into four categories based on ICM and TE score. Although poor-quality blastocysts were associated with an increased risk of SGA in another study (23), the findings for perinatal outcome of birthweight, PTD, LBW, and LGA, were not statistically significant. However, women with complications of pregnancy were not excluded in that study, which might explain the slight difference between the results of that study and our present findings.

The inconsistencies in the findings of the various studies in terms of perinatal outcomes may be caused by differences in inclusion and exclusion criteria, categories used to group blastocyst quality, and sample size distributions, especially the proportion of good-quality blastocysts in D6, as well as the relative subjectivity of blastocyst scoring. Pregnancy complications (31), uterine malformations (32), frozen-thawed technology (3), and different development rates from day 5 and day 6 (33), and the above-mentioned factors would all impact perinatal outcomes, including PTD. Meanwhile, another study (34) confirmed that an elevated AMH level would be an independent risk factor for PTD in overweight patients with polycystic ovary syndrome. Our study excluded cycles with the above factors, and only included frozen-thawed cycles with adjustment for confounding factors such as patient age and BMI, AMH level, and transfer day before performing multiple regression. Therefore, our findings can be considered reliable. Furthermore, it was reported in the literature (35) as early as 2016 that the incidence of spontaneous PTD in a community hospital decreased with a concomitant increase in the incidence of iatrogenic PTD, and that iatrogenic factors would contribute to the high rate of PTD (36). Given the lack of obstetric data in our dataset and interdisciplinary limitations, we could not separate iatrogenic preterm births, and the influence of iatrogenic preterm birth factors cannot be avoided.

The transfer of good-quality blastocysts has been associated with a higher likelihood of a male neonate (23), and this imbalance in sex ratio could be influenced by use of the intracytoplasmic sperm injection (ICSI) fertilization method (37). In a retrospective registry-based study in the UK (38), the male birth rate was 16% higher with ICSI cycles than with *in vitro* fertilization cycles. A retrospective study performed at 18 reproductive medicine centers in China (39) showed that use of ICSI reduced the secondary sex ratio in cleavage-stage embryo transfer cycles but not in blastocyst transfer cycles. At the same time, the male birth rate after SBT was positively correlated with TE grade, and blastocysts with TE grade A had a 2.53 higher probability of male sex than female sex (40). Therefore, we believe that TE could be a more predictable parameter for sex imbalance (41), but the mechanism involved is not yet precisely understood. The high male birth rate in expansion stage 6 is the result of rapid development of male blastocysts (42), while the decreased ratio in stage 5 would be related to the semi-hatching state. However, further studies are needed to explore the effect of expansion on the secondary sex ratio. Moreover, there is a trend toward sex-biased mortality during normal human development (38), with more significant mortality of female fetuses during the entire pregnancy. This mortality would be caused by the disrupted expression of maternally inherited mRNA or RNA synthesized, and the sex bias among normal/abnormal embryos was associated with the normal/abnormal state of the sex chromosomes and of chromosomes 15 and 17 (43, 44).

About 1.10%-1.75% of ART-conceived infants had at least one major birth defect (45). However, the infertile couples who achieve pregnancy by ART have the same risk of birth defects as couples who conceived naturally late; thus, some scholars believed that it was infertility itself but not the ART that resulted in the increased risk of birth defects (46). A multicenter report of birth defects among 15,405 Chinese offspring conceived by ART (47) found the incidence rate was between 1.11% and 1.58%, and infants born after IVF alone have the approximate incidence rate of birth defects compared to the general Chinese population. Due to the strict inclusion and exclusion criteria in this study, only 16 of the 2648 singleton live births were accompanied by birth defects, including circulatory, digestive, etc. There is no significant difference between the groups of single blastocyst transfer, but because of the small sample size, the results must be further verified.

Our study had several key strengths (1): a large sample size from a single center guaranteed the consistency of treatment and laboratory procedures, including the patient source, throughout the study period (2); limiting patient age and BMI excluded the influence of maternal age on blastocyst quality and avoided the influence of the woman’s weight on the birthweight of the neonate (3); multiple regression with adjustment for confounders, unadjusted and fully adjusted for multiple models, was used to confirm study findings (4); the correlation between morphological parameters of the blastocyst (including expansion stage, ICM, TE) and perinatal outcomes was observed in addition to the overall grading of blastocysts as good and poor quality; and (5) more perinatal outcomes were investigated than in previous studies. Based on the reference standard for birthweight in China, adjusting and classifying birthweight according to gestational age and neonatal sex (LGA, SGA) are more reliable than absolute birthweight analysis only.

One major limitation of this study is its retrospective nature and the relative subjectivity of blastocyst scoring based on morphological parameters. Additionally, good-quality blastocysts were preferred for transfer in view of patient requirements and the purpose of ART, and therefore were non-random. Furthermore, obstetric data, such as placental thickness and total gestational weight gain (48), are lacking in our database. A recent study (49) demonstrated a positive correlation between ultrasonographic placental thickness in the second trimester and birthweight.

Overall, our findings suggest that selective transfer of a single good-quality blastocyst can reduce the PTD rate but also results in a degree of sex bias in frozen-thawed cycles. We found that grade B ICM had the lowest PTD rate, and the higher the TE grade, the higher the probability of a male neonate. Blastocyst quality, including morphological grade, had no significant effect on length at birth, the risk of a birth defect, birthweight, LGA, and SGA.

## Data availability statement

The original contributions presented in the study are included in the article/supplementary material. Further inquiries can be directed to the corresponding author.

## Ethics statement

The studies involving human participants were reviewed and approved by Zhengzhou University and Henan Provincial People’s Hospital. This study did not require informed consent for participation following the national legislation and the institutional requirements.

## Author contributions

SZ supervised the entire study, including the procedures and design, and participated in revisions to the article. CZ participated in the study design and revised the article. NJ wrote the first draft of the manuscript. HH collected and analyzed the data. CZ and JX were responsible for the research conception. All authors reviewed and contributed to the manuscript and approved the submitted version.

## Acknowledgments

The authors are grateful to all the staff of the Reproductive Medicine Center, Henan Provincial People’s Hospital, for their support.

## Conflict of interest

The authors declare that the research was conducted in the absence of any commercial or financial relationships that could be construed as a potential conflict of interest.

## Publisher’s note

All claims expressed in this article are solely those of the authors and do not necessarily represent those of their affiliated organizations, or those of the publisher, the editors and the reviewers. Any product that may be evaluated in this article, or claim that may be made by its manufacturer, is not guaranteed or endorsed by the publisher.
